# Difficulties in the management of an Askin tumor in a pediatric patient with cystic fibrosis: case report and literature review

**DOI:** 10.3389/fped.2023.1289256

**Published:** 2023-12-01

**Authors:** Cristian Marinău, Andrei Csep, Cristian Sava, Alin Iuhas, Larisa Niulaș, Ariana Szilagyi, Ladislau Ritli, Andreea Balmoș, Claudia Jurca

**Affiliations:** ^1^ Faculty of Medicine and Pharmacy, University of Oradea, Oradea, Romania; ^2^ Bihor County Clinical Emergency Hospital, Oradea, Romania

**Keywords:** Askin’s tumor, Ewing’s sarcoma, pediatric cancer, cystic fibrosis, pseudomonas aeruginosa infection

## Abstract

Treating Ewing's Sarcoma of the thorax (Askin's tumor) with antineoplastic therapy in a malnourished cystic fibrosis patient colonized with Pseudomonas aeruginosa and Staphylococcus aureus may carry a significant potential for complications. We present the case of a known cystic fibrosis patient, diagnosed with Askin's tumor 5 years ago. Despite facing severe neutropenia, exacerbations of cystic fibrosis with Pseudomonas aeruginosa infections, and challenges in maintaining adequate caloric intake during the oncological treatment, the patient's outcome has been favorable. Chemotherapy doses had to be adjusted, and continuous antibiotic treatment was introduced throughout the course of therapy to reduce the frequency and intensity of exacerbations. Approximately 5 years after the cancer diagnosis, with no signs of relapse, the patient was started on CFTR (Cystic fibrosis transmembrane conductance regulator) modulator treatment. This intervention has successfully corrected the weight deficit. The coincidence of Ewing's sarcoma of the chest wall and cystic fibrosis in a single patient is 2.857 × 10^−5^% and to the best of our knowledge, this scenario has not been documented before.

## Introduction

The survival rates for patients with cystic fibrosis (CF) have consistently increased since the initial description of the disease in 1938, when the majority of patients did not live beyond their first year. By 2008, the median survival of CF patients in the UK reached 38.8 years. The enhanced survival in CF can be attributed to various interventions, including enhanced diagnostics and screening, improved nutritional care and access to pancreatic enzyme replacement therapies, the development of specific and specialized physiotherapy approaches, the concentration and specialization of CF treatment at regional centers, the availability and effective utilization of antibacterial therapies targeting Pseudomonas infections, and the use of CFTR (Cystic fibrosis transmembrane conductance regulator) modulators in disease management (1). Currently the predicted survival rate is at 65.6 years (2). As the survival age increases, associated conditions of various types, including cancer, arise, posing challenges in their management due to both the underlying pathology and the limited clinical experience and literature data regarding the association of these conditions.

Long-term cohort studies conducted across multiple centers have indicated a slight elevation in the risk of malignancy among individuals with CF. It is important to note that the overall incidence of cancer in CF patients remains relatively low, however, they do face an increased susceptibility to cancers affecting the digestive tract, especially after undergoing transplantation. Additionally, CF patients have a higher risk of developing lymphoid leukemia and testicular cancer, while their risk of melanoma appears to be reduced (3).

Ewing sarcoma, peripheral primitive neuroectodermal tumors, and Ewing's sarcoma of the thorax (also called Askin's tumor) are classified as part of the Ewing family of tumors, which exhibit varying degrees of neuroectodermal differentiation. Askin tumors specifically manifest as respiratory issues, including pain, dyspnea, and noticeable weight loss accompanied by the presence of a mass. These tumors are highly malignant, typically associated with a poor prognosis, and have a shorter overall survival rate (4).

We highlight the diagnostic and treatment challenges, and favorable outcome despite significant obstacles, of a case that presented to us: a cystic fibrosis patient, with chronic Pseudomonas aeruginosa and Staphylococcus aureus pulmonary infections and malnutrition who develops an Askin tumor.

## Case report

We describe the case of B.R., a 9-year-old girl, who is in our care since infancy.

The girl is the first child of the family, born to healthy parents, from a normal pregnancy, delivered spontaneously at full term, weighing 2,700 grams, with good adaptation to extrauterine life, and an Apgar score of 9/10. Newborn screening for cystic fibrosis was not performed.

At the age of 3 months, after a persistent bronchopneumonia, chronic diarrhea, and failure to thrive, a suspicion of cystic fibrosis arose. This was later confirmed by two positive sweat tests and through complete sequencing of the CFTR gene, revealing the mutations c.54_273del (exons 2 + 3) and c.1521_1523del (exon 11), leading to the diagnosis of compound heterozygous cystic fibrosis, with the ΔF508 variant in its complete clinical form. While the ΔF508 genotype is associated with pancreatic insufficiency, neither fecal elastase nor the 72-hour stool fat were measured. However, fat globules were visualized by microscopy, prompting the initiation of pancreatic enzyme replacement therapy, which improved the steatorrhea. No evidence of CF hepatobiliary disease was found during clinical assessment, in blood work, or on liver ultrasound. The child was started on a comprehensive treatment regimen including medications to facilitate mucus clearance (daily Dornase alpha and hypertonic saline nebulization), pancreatic enzyme replacement therapy (Kreon – 10,000 UI with each meal) and vitamin (A, D, E, K) supplements, probiotics. Additionally, airway clearance techniques were incorporated into the daily management plan. Also at this age, the child was diagnosed with Pseudomonas aeruginosa infection (confirmed by pharyngeal aspirate culture) and Staphylococcus aureus infection (confirmed by catheter tip culture).

From the age of 3 months until 4 years, the child experienced multiple hospitalizations due to metabolic alkalosis, hyponatremia, and hypokalemia, leading to the diagnosis of Pseudo-Bartter syndrome. Additionally, during this period, the child had repeated respiratory infections with Pseudomonas aeruginosa and Staphylococcus aureus, and was diagnosed with protein-calorie malnutrition.

**Figure 1:**
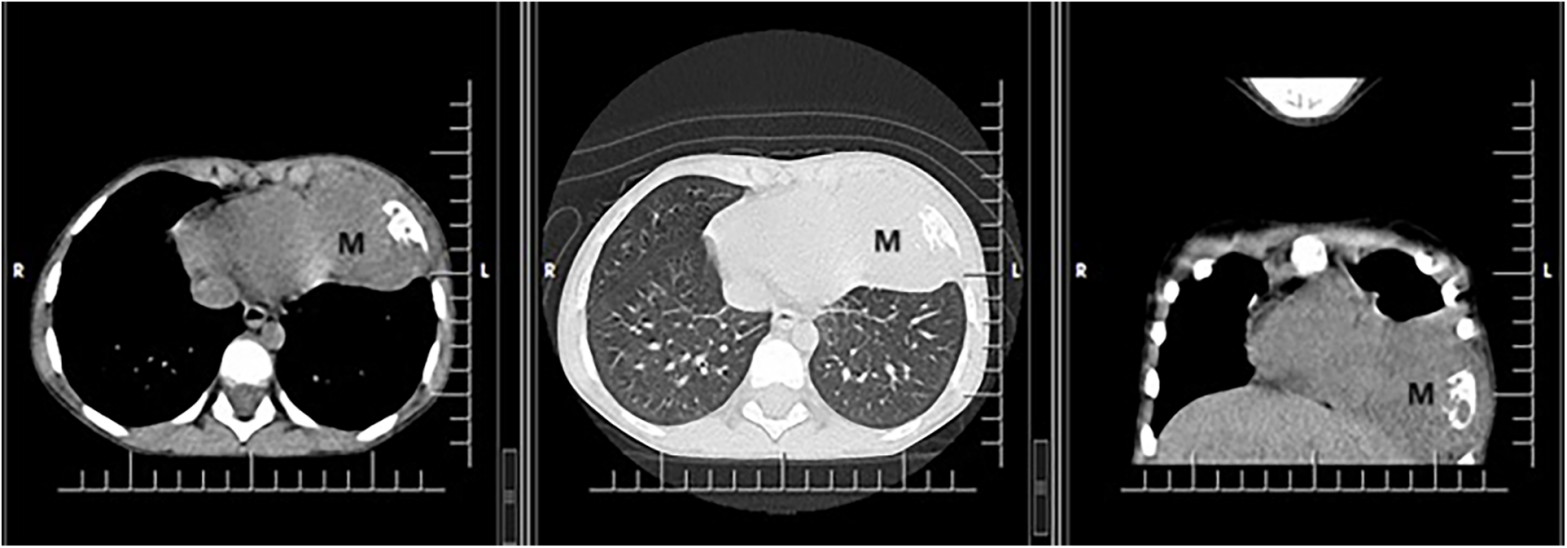
CT scan showing a voluminous parenchymal mass (M) of the anterior left thoracic wall, relatively well delimited, with bone destruction, which invades the lung parenchyma up to the level of the heart, with mass effect on left ventricle.

In October 2018, at age 4, during an episode of acute respiratory infection, bilateral basal pulmonary consolidation processes were observed, more significant on the right side. The condition improved with antibiotic treatment for the left-sided lesion, but the right-sided lesion persisted. A follow-up chest CT scan performed after 14 days of treatment, revealing a parenchymal lesion measuring 61/56/39 mm with aggressive features (osteolysis and pericardial reaction) located at the base of the left hemithorax (Figure 1).

**Figure 2:**
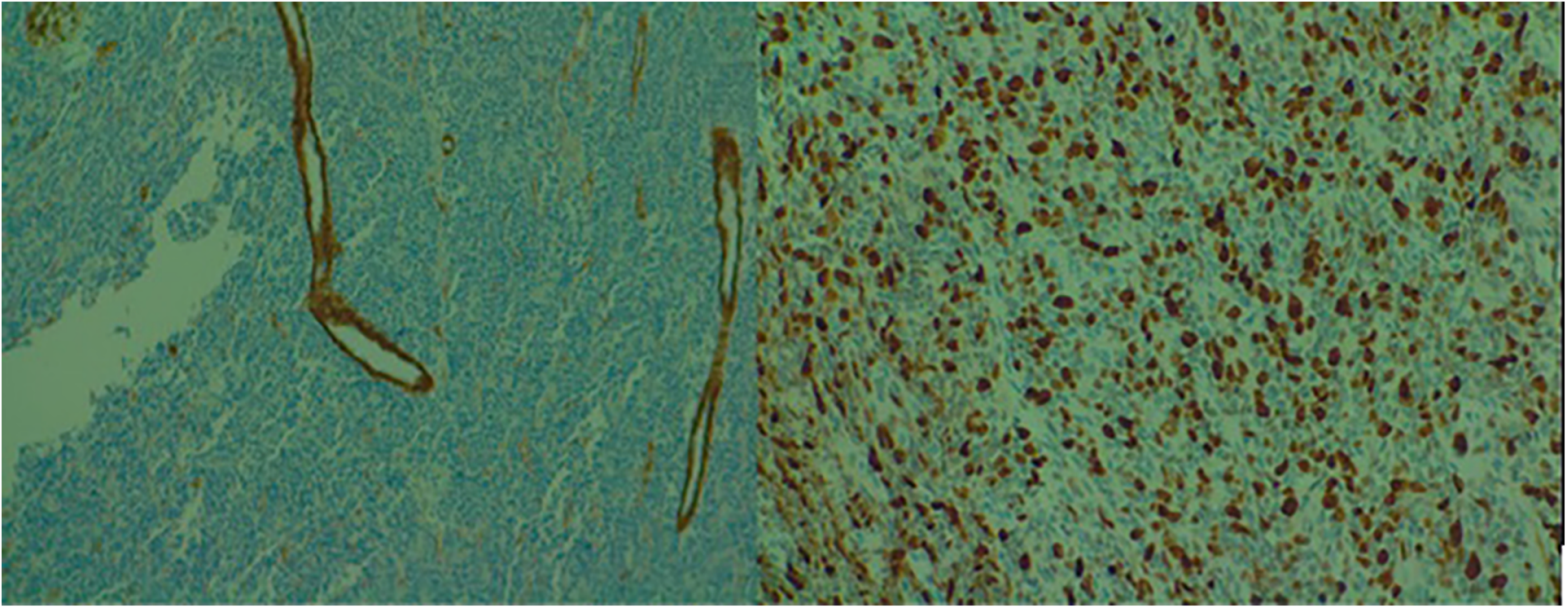
Microscopic examination of the biopsied lesion displaying small round to oval cells with dispersed chromatin [(**A**) 10× and (**B**) 40× magnification].

A biopsy of the lesion (rib and soft tissue) was performed, showing histopathological features consistent with a malignant proliferation of small round-oval cells with dispersed chromatin. Immunohistochemistry (IHC) tests were positive for CD99 and negative for CD56, S100, chromogranin A, muscle actin, CD45, and CD34. Ki-67 proliferation index was 60%. FISH testing revealed rearrangements of the EWSR1 gene (22q12) in 65% of tumor cells, leading to the conclusion of Ewing/PNET sarcoma, with architectural and localization characteristics of an Askin tumor (Figure 2).

**Figure 3:**
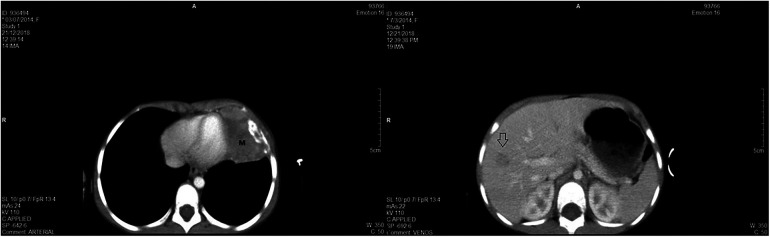
(**A**) - CT scan revealing voluminous parenchymal mass (M) of the anterior left thoracic wall, relatively well delimited, with bone destruction, which invades the lung parenchyma up to the level of the heart, with mass effect on left ventricle; and (**B**) - hypodense formation in the segment VII of the liver.

Prior to the treatment course, the case was reevaluated through imaging. An 85 × 60 × 35 mm formation in the lower half of the left hemithorax, with a tumor volume of 140.1 cm^3^, and a hypodense formation of approximately 15 mm in segment VII of the liver were identified (Figure 3).

Chemotherapy treatment was initiated consisting of 6 cycles of VIDE (Vincristine 1.5 mg/m^2^/d, day 1; Ifosfamide 3.0 g/m^2^/d, day 1, 2, 3; Doxorubicin 20 mg/m^2^/d, day 1, 2, 3; and Etoposide 150 mg/m^2^/d, day 1, 2, 3), at 21 days intervals, followed by surgical removal of the residual tumor and 8 cycles of VAI (Vincristine 1.5 mg/m^2^/d, day 1; Actinomycin D 0.75 mg/m^2^/d, day 1, 2; Ifosfamide 3.0 g/m^2^/d, day 1, 2) at 21 days intervals, according to EURO-EWING 99 protocol. The child showed favorable evolution of the oncological disease, with the tumor shrinking by 88% to 16.5 cm^3^ after 2 cycles of VIDE, and further reducing to 9 cm^3^ after 4 cycles. The hepatic lesion also decreased in size from 15 mm to 8 mm in diameter. Hematological recovery was obtained on average after 5 days of substitutive and supportive treatment, but with each aplasia episode, pulmonary decompensation occurred due to bacterial overgrowth, leading to life-threatening conditions (marked respiratory distress, tachypnea, oxygen dependency) and causing delays in the chemotherapy protocol (3th VIDE cycle delayed by 7 days, 4th VIDE cycle delayed by 11 days, 5th VIDE cycle delayed by 16 days).

Initially, antibiotic treatment was administered only during infectious exacerbation periods with systemic antipseudomonal antibiotics in usual, therapeutic doses (carbapenems – Meropenem 20 mg/kg/dose i.v. every 8 h; aminoglycosides – Amikacin 6 mg/kg/dose i.v. every 8 h; fluoroquinolones - Ciprofloxacin 10 mg/kg/dose i.v. every 8 h; or oxazolidinones – Linezolid 10 mg/kg/dose i.v. every 8 h). During one of these pulmonary decompensations a right-sided basal consolidation adjacent to an expanding bronchial path was identified. Culture of sputum, lavage, and tracheal aspirate revealed once again Pseudomonas aeruginosa. After this, concomitant with the 5th VIDE cycle, continuous intravenous (antipseudomonal agents in usual doses) and nebulized antibiotic therapy (Tobramycin 300 mg and/or Colistin 1,000,000 UI aerosols at 12 h intervals) antibiotic therapy was introduced in an attempt to reduce Pseudomonas aeruginosa and Staphylococcus aureus colonies Additionally, the dosage of Etoposide was reduced by 20%. These measures led to a decrease in the severity of exacerbations.

After 6 cycles of VIDE chemotherapy, surgical intervention was performed, carrying out a left thoracotomy in the 6th intercostal space. The remaining tumor formation is resected, along with the resection of the sixth and seventh rib, and an atypical left lower lobe lobectomy. The hepatic metastasis was not detected. The histopathological result reveals the presence of tumor cells with post-therapeutic reactive changes, which constitute less than 5% of the tumor volume (Salzer-Kuntschik: grade 2 histopathological response). Pseudomonas aeruginosa is isolated once again from the culture obtained from the tip of the orotracheal intubation tube. However, the patient's condition continues to improve under antibiotic treatment (Ceftazidime 50 mg/kg/dose i.v. every 8 h and Colistin nebulization), and she is retransferred to the oncology department for further oncological treatment. Complete response was declared at this point (June 2019), along with the complete disappearance of all visible disease. The treatment is continued with 8 cycles of consolidation chemotherapy with VAI, which, along with continuous treatment with Colistin aerosols and supportive care, is well tolerated.

Throughout the therapy, we have also faced feeding issues as the child, who already had weight deficit and pseudo-Bartter syndrome, refuses oral feeding, necessitating nasogastric tube feeding and parenteral nutrition.

Regular follow-up examinations have been conducted, and up to the present - 5 years from the initial diagnosis, no disease recurrence has been detected.

**Figure 4:**
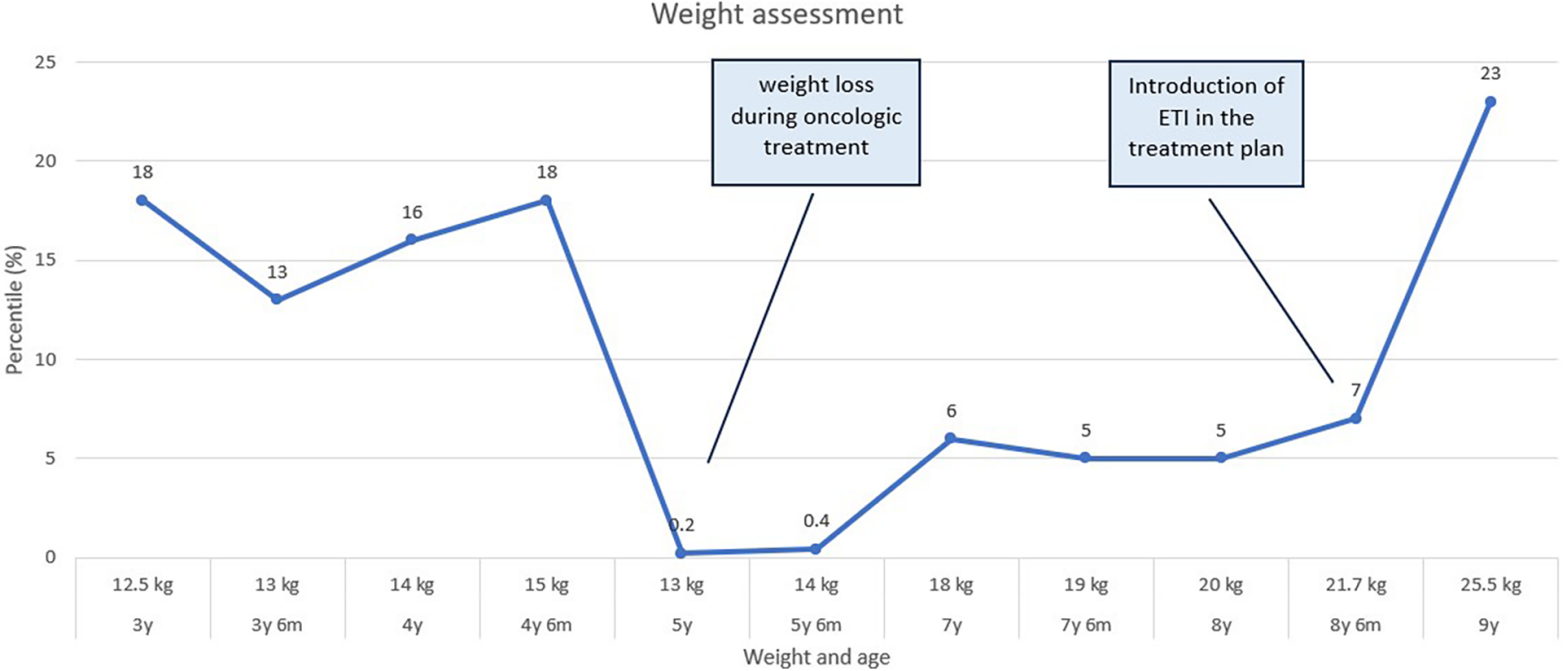
A graph depicting the child's weight curve over the past 6 years reveals a notable weight loss during the oncological treatment period, and significant increase upon the initiation of EIT (elexacaftor/tezacaftor/ivacaftor) treatment.

In March 2023, CFTR modulator therapy with Elexacaftor/Tezacaftor/Ivacaftor (ETI) was initiated and has been well-tolerated, resulting in a remarkable improvement in the weight curve (Figure 4). However, despite achieving adequate weight gain, there is persistent pulmonary colonization with Pseudomonas aeruginosa, necessitating ongoing treatment with inhaled and systemic antibiotics, as well as mucolytic medication.

## Discussions

Chest wall tumors in children and adolescents are uncommon, occurring with an incidence of 1 in 1,000,000 (5). Basharkhah et al. (6) report a five-year survival rate of 86% in a group of patients with Askin tumors from a single center, with an overall survival rate of 71%; the authors report significantly improved prognoses compared to Contosso et al. (7)., a study conducted a decade earlier, when the prognosis is generally unfavorable, with survival rates at 2 and 6 years being 38% and 14% respectively. The Basharkhah et al. reports similar values to those cited in acute lymphoblastic leukemia in children (8). While the majority of Askin tumors are situated in the ribs, instances of localization in the paravertebral, sternal, and scapular regions have been documented (9). Some tumors have also been found in the lung (10).These tumors often become apparent relatively late, typically following considerable local enlargement of the tumor or when presenting as a painful mass; as a result of their delayed appearance, some patients present with metastases or malignant pleural effusion. Imaging studies commonly reveal bone destruction in the affected rib, and a substantial extension of the tumor mass into neighboring regions (6). Askin tumors exhibit a high degree of aggressiveness. They primarily manifest in children and adolescents, although they can develop at any age, with a notable predominance among females (75% of cases) (11, 12).

Cystic fibrosis stands as the most prevalent autosomal recessive disorder within the Caucasian population, with an incidence of roughly 1 in 3,500 births (13). The majority of patients display symptoms either at birth or shortly thereafter, with respiratory infections and inadequate weight gain being the most common initial presentations (14). Numerous countries have implemented newborn screening for CF due to the fact that early diagnosis enhances treatment outcomes (15). For our patient, screening wasn't conducted at birth; during that period in Romania, newborn screening through national program only included phenylketonuria and congenital hypothyroidism (16).

As individuals with CF experience increased longevity new risks such as cancer emerge. Although the overall occurrence of cancer in individuals with CF is relatively low, they do exhibit a heightened vulnerability to cancers affecting the digestive tract, particularly following transplantation (17). Adult patients with cystic fibrosis are markedly vulnerable to the premature and aggressive development of colorectal tumors; by the age of forty, 50% of individuals with CF will develop adenomas, of which 25% will progress to advanced aggressive adenomas, and a portion of these may have already transitioned to adenocarcinomas (18). The incidence of colorectal cancer in adults with CF is 5–10 times higher than that in the general population. Additionally, after undergoing an organ transplantation, CF patients face a significantly elevated risk due to immunosuppressive therapy, which is 25–30 times greater (19).

While literature extensively covers digestive system cancers in patients with cystic fibrosis, reports describing other types of cancers are scarce. And very few of these are in pediatric cases.

A case series describes 11 patients with cystic fibrosis that were diagnosed with a malignant disease (20). Among these are four cases of gynecological malignancies (including cervical intraepithelial neoplasia and cervical cancer), two instances of hematological malignancies (acute lymphocytic leukemia), one case of gastrointestinal malignancy (peritoneal mesothelioma), and four occurrences of malignancies originating from various sources (such as malignant melanoma, neuroblastoma, adrenocortical carcinoma, and thyroid cancer). Three patients were pediatric patients: a 9-month-old girl with neuroblastoma, 14-year-old girl with acute lymphocytic leukemia and another 17-year-old girl with acute lymphocytic leukemia. The first patient died three years after the initial diagnosis, after a systemic relapse and a pulmonary exacerbation which forced the discontinuation of chemotherapy; the two leukemia patients died within months from the initial diagnosis of malignancy due to infections complications.

Okuda et al. (21) and Ruffles et al. (22) describe two cases of osteogenic sarcomas in patients with a similar background to our pediatric cystic fibrosis patient. In the first case the patient was colonized intermittently with typical bacteria, such as Staphylococcus aureus, Haemophilus influenza, Serratia marcescens, and Escherichia coli, and in the second case the patient had a chronic Pseudomonas aeruginosa infection. In the case presented by Okuda et al., during neutropenic phases, prophylactic intravenous cefuroxime was given. Two febrile neutropenic episodes were managed with antibiotics following standard protocols. The authors concluded that the antineoplastic treatment was well-managed without worsening of CF lung disease. Similarly, in the second case, severe complications of immunosuppression were prevented, despite the chronic infection with Pseudomonas aeruginosa.

To the best of our knowledge, no other association between cystic fibrosis and tumors from the Ewing sarcoma family in a pediatric patient has been described in the existing literature. Taking into consideration the incidence of Ewing's sarcoma of the chest wall and the incidence of cystic fibrosis, it is possible to calculate, by multiplying these individual events, the chance of the occurrence of this disease association in a patient. This is 2.857 × 10^−5^%.

Our case provides valuable insights as the patient encountered and overcame distinct challenges associated with the persistent presence of Pseudomonas aeruginosa and Staphylococcus aureus in her sputum and feeding issues during her treatment. Fatal outcomes have been documented for CF patients due to hemorrhagic bronchopneumonia resulting from Pseudomonas aeruginosa infection during immunosuppression (23). The medical care of such a patient necessitated the attention of a multidisciplinary team whose collaboration enabled the overcoming of challenges that arose during the course of oncological treatment.

In our case, during the neutropenic phases after chemotherapy, exacerbations of cystic fibrosis with Pseudomonas infections led to life-threatening situations. Antipseudomonal agents were used according to standard protocols; however, these exacerbations delayed the administration of VIDE cycles according to the protocol. In an attempt to reduce treatment toxicity, the dose of Etoposide was decreased by 20%, which was allowed by the oncologic protocol. After multiple exacerbations, the need for preventing them became evident. A continuous treatment with anti-pseudomonal antibiotics was initiated, both intravenously and through aerosol administration. These measures, combined with the standard treatment for cystic fibrosis, respiratory physiotherapy, and mucolytics, led to significantly reduced intensity of exacerbations. This improvement allowed adherence to the required timelines dictated by the oncologic protocol.

While treatment for Askin's tumor lacks standardization (24), the majority of authors recommend a combination of neoadjuvant chemotherapy, radiotherapy, and surgical resection as the most suitable and adaptive treatment measures (12). The absence of clinical guidelines that establish standardized management contributes to an unfavorable prognosis and a limited survival rate (24) In our patient's case, initial tissue biopsy was followed by neoadjuvant chemotherapy before surgical resection. This approach is supported by Sirivella et al. (25), whose report suggests that the implementation of neoadjuvant chemotherapy led to an improvement in the 10-year survival free of disease indicator, observed in up to 84% of patients without metastases. Surgical resection is essential, and neoadjuvant chemotherapy leads to negative margins in 71% of cases, compared to 37% when surgical resection is the initial approach (26). Although most authors recommend it (24, 27), radiotherapy was not employed in our patient's case. Considering the complete resection with clear margins of the tumor, as well as the known pulmonary toxicity associated with both radiotherapy and certain chemotherapeutic agents like vincristine (28, 29), and taking into account the pre-existing lung disease, radiotherapy was not deemed suitable for our patient.

The weight deficit reached during the oncological treatment persisted for an extended period, with a noticeable improvement becoming evident only recently, after the initiation of CFTR modulator treatment. This period partially overlaps with the COVID pandemic period, which led to reduced patient-doctor interactions due to the limited contact opportunities. This issue becomes evident at all levels, particularly affecting patients with chronic conditions (30). The initiating of ETI therapy, alongside more effective monitoring of the patient's disease progression, has led to an improvement in the overall condition, with fewer pulmonary exacerbations and a favorable trend in the weight curve, with no signs of modulator-induced liver injury. Unfortunately, the lack of an initial fecal elastase level prevented us from tracking any enhancements in pancreatic function after 12 months of ETI therapy.

Adverse effects on breast tissue have been noted with the use of CFTR modulators, primarily among adult patients. Breast-related issues such as breast masses, breast inflammation, gynecomastia, and nipple disorders have been reported in both genders. A case report illustrated an unusual dose-dependent side effect of breast development in a 7-year-old girl with CF who was undergoing treatment with ivacaftor (31).

Up until the time of publication of this article there is a reassuring absence of any signs of cancer relapse. However, despite achieving adequate weight gain, there is persistent pulmonary colonization with Pseudomonas aeruginosa, necessitating ongoing treatment with inhaled and systemic antibiotics, as well as mucolytic medication and nutritional support.

The incidence of cancer is anticipated to rise in the coming years, in tandem with the aging of the cystic fibrosis population and the growing number of adults with cystic fibrosis who have undergone lung transplantation. The impact of CFTR modulators on cancer risk remains uncertain and will necessitate further evaluation (32).

The strengths of this study lie in its capacity to illuminate a rare and complex clinical scenario, serving as a foundational platform for more rigorous research. This case report underscores the significance of tailored, patient-centric oncologic care, particularly for this susceptible patient demographic. However, the study is constrained by the inherent limitations of any single-patient case reports. The insights and experiences of the individual patient may not be broadly generalizable to a larger population, and establishing causality between the observed outcomes and the treatment or other factors is challenging. Furthermore, with respect to the CFTR modulator treatment, while the initial results are encouraging, the observation period for its effects has been relatively short, a more extended period of observation is essential to draw conclusive insights.

## Data Availability

The datasets presented in this study can be found in online repositories. The names of the repository/repositories and accession number(s) can be found in the article.
